# Effect of metabolic activity of lactic acid bacteria and propionibacteria on cheese protein digestibility and fatty acid profile

**DOI:** 10.1038/s41598-023-42633-w

**Published:** 2023-09-16

**Authors:** Małgorzata Ziarno, Joanna Bryś, Ewa Kowalska, Patrycja Cichońska

**Affiliations:** 1 Institute of Food Sciences, Department of Food Technology and Assessment, Division of Milk Technology, Warsaw University of Life Sciences, Warsaw, Poland; 2 Institute of Food Sciences, Department of Chemistry, Warsaw University of Life Sciences, Warsaw, Poland

**Keywords:** Applied microbiology, Nutrition

## Abstract

This study aimed at investigating the influence of different variants of bacterial starter cultures on the metabolism of the bacteria used, cheese protein digestibility, and fatty acid profile. The results revealed that lactic acid bacteria had a significant effect on the proportions of fatty acids in cheeses, with saturated fatty acids being predominant in in all cheese variants. Fatty acid proportions are complex and depend on the type of cheese culture and monoculture used. Additionally, the analysis of fatty acid composition showed variations in the proportion of saturated and unsaturated fatty acids, impacting the values of atherogenic and thrombogenic indices. Notably, the atherogenic index was highest in samples of mature cheeses obtained from a typical mesophilic cheese culture, whereas it was lowest in samples of fresh milk and mature cheeses obtained from a mesophilic cheese culture and monocultures of *Lacticaseibacillus casei* and *Propionibacterium*. The study also highlighted the influence of lactobacilli on the content of available free lysine, glycine, and methionine in cheese proteins. Mature cheeses obtained with *Propionibacterium* and *L. casei* starter cultures exhibited higher free lysine and glycine content compared with fresh cheeses and those obtained solely with the cheese culture. Additionally, mature cheeses obtained with starter cultures of mesophilic cheese culture, *Propionibacterium*, and *L. casei* had the highest free methionine content. Based on these findings, it is evident that the choice of cheese making cultures and monocultures can significantly affect the fatty acid composition and amino acid content of cheese and fresh milk, potentially bearing important health implications.

## Introduction

As the focus on maintaining a healthy and balanced diet continues to gain popularity, consumers are increasingly seeking accurate nutrient analyses and understanding their true value. Among the key components of a well-rounded diet, protein stands out as one of the most essential elements proper body functioning, since it is an essential component of most processes that occur in the body^1–4^. Digestibility, together with essential amino acid composition, is a determining factor in assessing the nutritional value of protein^5,6^. Additionally, related to this is digestibility, which refers to the susceptibility of components to digestive enzymes and the extent to which the resulting broken-down components can be effectively utilized^7^. Therefore, understanding the content of what is being consumed becomes paramount in developing a well-balanced diet and ensuring the consumption of high-good quality food, that not only appeals to taste but also holds functional values for the consumer's body. Numerous studies have delved into food processing and its impact enhancing the quality and digestibility of proteins^8–10^.

Beyond proteins, fats stand as another crucial nutrient group in our diet. In the context of fermented products, both long- and short-chain fatty acids present therein hold significant value. The key lies in understanding their interrelationship and proportions within the total content, necessitating the need for a more comprehensive analysis of their effects on the consumer's body^11^. The proportions of fatty acids hold promise not only as a subject of research for a balanced diet, but also for the study of triacylglycerols present in these products. Differences in structure lead to noticeable distinctions in the rate of metabolization and sensory characteristics of the final products. Furthermore, the interaction between proteins with fats, particularly their oxidation products, emerges as a factor impacting the nutritional value of proteins^12^. Lipid oxidation products can also lead to a reduction in essential enzymes, such as trypsin, which plays a crucial role in food digestion^13^.

Among dairy products, rennet-ripened cheeses hold immense significance due to their high protein and fat content. Milk proteins offer a diverse and abundant amino acid composition, making them a valuable source of nitrogen, essential for bacterial growth^14^. In the production of these cheeses, *Lactococcus* bacteria, particularly the species *Lactococcus lactis* subsp. *lactis,* play a crucial role. To achieve the desired flavor and characteristics, additional strains of microorganisms are introduced alongside the starter bacteria, depending on the type of cheese being produced. For instance, cheeses like Dutch-type (such as Edam-type or Gouda-type) and Swiss-type (like Emmental-type) are produced by incorporating bacteria from the species such as *Streptococcus thermophilus*, *Lactobacillus delbrueckii* subsp. *bulgaricus*, *Lactobacillus delbrueckii* subsp. *lactis, Lactobacillus helveticus, Lacticaseibacillus casei,* and bacteria from the genus *Propionibacterium*^15–17^.

The objective of this study was to investigate the impact of various bacterial starter cultures, including lactic acid bacteria and propionibacteria, that can be used in the production of rennet-ripened cheeses, on cheese protein digestibility and fatty acid profile. The results can be utilized in the dairy industry to create valuable products for consumers, focusing on improving both protein digestibility and the quality of fat, thereby enhancing diets and promoting better health for people.

## Materials and methods

### Materials and reagents

The raw materials used to obtain the cheese samples adhered to the requirements stipulated in Regulation (EC) No 853/2004 of the European Parliament and of the Council, dated 29 April 2004, which lays down specific hygiene rules for food of animal origin^18^. For coagulation, the enzyme Fromase^®^ 2200 TL Granulate (DSM, Delft, The Netherlands) was used, diluted with tap water at a ratio of 1:20. The study employed the following three industrial freeze-dried starter cultures: F-DVS Flora Danica, representing a typical mesophilic cheese culture type LD, consisting of a blend of strains from species such as *Lactococcus lactis* subsp. *cremoris*, *Lactococcus lactis* subsp. *lactis, Leuconostoc mesenteroides* subsp. c*remoris,* and *Lactococcus lactis* subsp. *diacetylactis*, provided by Chr. Hansen, Cząstków, Poland). Additionally, a mesophilic dairy monoculture, F-DVS L. casei-01, containing *Lacticaseibacillus paracasei,* also supplied by Chr. Hansen. The third starter culture, PROPIONICI (by Dalton Biotecnologie S.r.l., Villa Raspa, Italy), was a dairy monoculture comprising *Propionibacterium freudenreichii* subsp. *shermanii*, generously provided by AGM Innotech Sp. z o.o. (Kalisz, Poland). Calcium chloride solution, serving as an additive to cheese milk, was of chemical grade (Browin, Łódź, Poland), diluted with distilled water at a ratio of 1:5. The sodium chloride used for salting the cheeses was of food-grade quality. Chemical reagents employed in the chemical analysis were of analytical grade and were obtained from local suppliers.

### Obtaining cheese samples in laboratory settings

To produce the cheese samples, raw cow milk was sourced from a private dairy farm near Warsaw (Poland, in two independent batches of 10 L each). The average composition of the milk was found to be 3.6 ± 0.1% protein, 3.8 ± 0.2% fat and 12.8 ± 0.1% dry matter, with an average pH value of 6.66 ± 0.01. The milk underwent a series of meticulous steps: it was batch skimmed at 47 °C using a disc separator and then pasteurized in a tank at 63 °C for 30 min. before being cooled to reach the ideal cheese-processing temperature of 30 °C. The cheese milk produced in this manner had calcium chloride added at a concentration of 0.02% (w/w). Thorough stirring ensued and after 15 min., either the mother culture (prepared from the F-DVS Flora Danica starter in a small portion of milk at 30 °C for 20 h) was added at the rate of 1.5% of the amount of processed milk or a dose of additional starter (F-DVS L. casei-01 or PROPIONICI diluted in a small portion of milk immediately before use) was added at the same time at a rate of 0.1% of the amount of processed milk. The mixture was allowed to sit for 40 min. After this time, a 0.02% (v/w) portion of rennet was added and the milk with additives was stirred for 1 min., then kept at 30 °C until it coagulated. Once the curd reached the desired compactness, it was cut using a harp and a lyre into grains with specific edge lengths of 4 and 11 mm, respectively, corresponding to the grain size of curd in hard and semi-hard cheeses. To achieve the appropriate grain firmness and elasticity were achieved, the resulting curd was stirred for 5–10 min. Subsequently, 40% (w/w) of the whey was drained, and rinse water (previously pasteurized and chilled to 32 °C) was added to 40% of the remaining cheese curd. After 5 min. drying period, the curd was reheated to 37 °C, ensuring that the temperature increase did not exceed 1 °C in 2 min. After reaching the set temperature, the curd was dried for 15–20 min. The resulting curd mass was then transferred into prepared cheese molds and prepressed in a cheese press to eliminate part of the whey. Each prepressed cheese was weighed and the pressure on the cheese press was calculated (20 per 1 kg of cheese mass). Pressing lasted approximately 5 h at 18–20 °C. Subsequently, the cheeses were removed from the molds, inverted, and placed back the molds for an additional 6 h at 18–20 °C. After this time, the cheeses were salted in a pre-prepared brine (18% (w/w) aqueous sodium chloride aqueous solution) at 12 °C. Each cheese was immersed in the brine for 24 h at 12 °C and then drained for an additional 24 h in a cheese ripening chamber at 12 °C and approximately 80% humidity. On completion of this process, the cheeses were divided into 120-g pieces, each wrapped in ripening film (BK4L, Cryovac, Sealed Air Polska Sp. z o.o., Duchnice, Poland) and packed under atmospheric pressure (999 mbar, at 1.5 min) using a Multivac (MULTIVAC Poland, Natalin, Poland) before being placed in the cheese-ripening chamber.

Similarly, rennet cheese models were produced in laboratory settings using industrial cultures of lactic acid bacteria or propionic acid bacteria in the following variants:

- KM samples using F-DVS Flora Danica,
- KMC samples using F-DVS Flora Danica + F-DVS L. casei-01,
- KMP samples using F-DVS Flora Danica + PROPIONICI,
- KMCP samples using F-DVS Flora Danica + F-DVS L. casei-01 + PROPIONICI.

The cheese samples underwent a 6-week aging process in a cheese-ripening chamber maintained at 12°C with approximately 80% humidity. At both the fresh stage and after six weeks of ripening, samples from each cheese variant were subjected to thorough chemical and microbiological analyses. Furthermore, the presence of molds, yeasts, and bacteria from the *Enterobacteriaceae* family was assessed in the cheese samples. The experiment was conducted twice.

### Microbiological analyses

To enumerate total lactococci cells, M17 Agar (Merck, Darmstadt, Germany) was prepared according to the manufacturer's instructions. The M17 agar plates were incubated at 30 °C for 72 h under aerobic conditions. For the determination of *L. casei* cells, MRS Agar (Merck, Darmstadt, Germany) was prepared as per the manufacturer's instructions. The MRS agar plates were incubated at 37 °C for 72 h under anaerobic conditions. The viable population of *Propionibacterium* was determined using *Propionibacterium* agar prepared following the method described by Atlas^19^. The *Propionibacterium* agar medium contained casein peptone (10.0 g/L), tryptic digest (10.0 g/L), sodium lactate (10.0 g/L), yeast extract (5.0 g/L) and agar (15.0 g/L), with a fixed pH range of 7.0–7.2 at 25 °C. The *Propionibacterium* agar plates were incubated at 37 °C for 7 days under anaerobic conditions. To detect *Enterobacteriaceae* cells, VRBD Agar (Merck, Darmstadt, Germany) was prepared according to the manufacturer's instructions. The VRBD agar plates were incubated at 30 °C for 48 h under anaerobic conditions. For the determination of mold and yeast populations, YGC Agar (Merck, Darmstadt, Germany) was prepared according to the manufacturer's instructions. The YGC agar plates were incubated at 25 °C for 5 days under aerobic conditions. All analyses were conducted on six independent samples, and the results were expressed as colony-forming units (CFU) per 1 g of cheese sample.

### Determination of water content

For the determination of the total water content, the study was performed in accordance with ISO 5534:2004 | IDF 4:2004 standard. Cheese and processed cheese—Determination of the total solids content (Reference method)^20^. The analysis was conducted on six independent samples, and the results were recorded with an accuracy of 0.01 g.

### Determination of total protein content

The study was carried out following the guidelines specified in ISO 1871:2009. Food and feed products—General guidelines for the determination of nitrogen by the Kjeldahl method^21^. The analysis was conducted on six independent samples, and the results were recorded with an accuracy of 0.01 g.

### Determination of fat content

The study was conducted following the guidelines outlined in ISO 3433:2008 | IDF 222:2008. Cheese—Determination of fat content—Van Gulik method^22^. The analysis was conducted on six independent samples, and the results were recorded with an accuracy of 0.01 g.

### Determination of chloride content

The study was conducted following the guidelines outlined in ISO 5943:2006 | IDF 88:2006. Cheese and processed cheese products—Determination of chloride content—Potentiometric titration method^23^. The analysis was conducted on six independent samples, and the results were recorded with an accuracy of 0.01 g.

### The pH measurement

Measurements were performed using a CPO-505 pH meter (Elmetron, Zabrze, Poland). The cheese samples, finely ground and mixed with distilled water in a 1:1 ratio, were filled into a small beaker. The pH meter electrode was immersed in the sample, and the reading was made with an accuracy of 0.01. The analysis was conducted on six independent samples, and the results were recorded with an accuracy of 0.01 g. The pH-meter was calibrated according to the manufacturer's instructions before use.

### Determination of protein digestibility

Protein digestibility was determined using three methods. The first method was described by Hęś et al.^24^, which involves determining the content of free lysine to assess the digestibility of protein^24,25^. Briefly, the principle of the first method involves reacting trinitrobenzene sulfonic acid (TNBS) with the free amino groups of lysine after prior acidic hydrolysis of the protein. The concentration of free lysine is then determined spectrophotometrically 415 nm relative to a standard curve using DL-lysine monohydrochloride as the standard. Available lysine content was determined using the method described in the study by Hall et al.^26^ and was expressed as mg per 100 g of cheese sample. The analysis was conducted on six independent samples. The second method, described by Pokorska-Lis et al.^27^ consists of subjecting the test samples to a two-step enzymatic hydrolysis process. First, pepsin digestion is carried out in a hydrochloric acid environment, followed by trypsin digestion in a slightly alkaline environment. The concentration of available free glycine is then determined spectrophotometrically at 570 nm by reaction with ninhydrin and comparing it against a standard curve. The content of available free glycine was expressed as mg/100 g of cheese sample and the analysis was conducted on six independent samples. The third method, described by Hęś et al.^24^ involves the determination of free methionine by using conducting an enzymatic protein hydrolysis. After the enzymatic hydrolysis, sodium nitroprusside was added to detect the sulfonic groups of the free methionine. Spectrophotometric measurements were taken at 520 nm with DL-methionine used as the standard. The content of free methionine was expressed as mg/100 g of cheese sample, the analysis was conducted on six independent samples.

### Determination of the fatty acid profile

For the extraction of the lipid fraction from cheese samples, the Folch method modified by Boselli et al.^28^ was used. The analysis was conducted on six independent samples. Prior to analysis, the analyzed fat samples were converted to fatty acid methyl esters, according to the method described in Ref.^29^. Gas chromatography using the YL Clarity chromatograph, equipped with a flame ionization detector and a capillary column with a BPX 70 packing—with a length of 60 m, a diameter of 0.25 mm, and a film thickness of 0.25 μm—was employed to analyze the fatty acid content. During the operation of the device, the temperature profile increased from the initial temperature of 60 °C and gradually increased by 10 °C/min until reaching 180 °C. Subsequently, there was a temperature jump to 230 °C with an increase of 3 °C/min., and the set temperature was held for 15 min. The injector and detector temperatures were maintained at 225 °C and 250 °C, respectively. The total analysis time for each sample was precisely 52 min. To determine the fatty acid content, an available standard with known retention times was used for calibration. The areas of the peaks in the chromatogram corresponded to the quantitative content of fatty acids. Each sample was analyzed twice, and the final average value was calculated for accurate results.

### Calculation of atherogenic and thrombogenic indices

To evaluate the nutritional quality of cheese samples, two important indices, the atherogenic index (AI), which is an indication of the tendency to produce microcoronary and macrocoronary diseases, and the thrombogenic index (TI), which was an indicator of the tendency to form clots in the blood vessels, were calculated according to Eqs. (1) and (2)^30–33^.

1 $$\mathrm{AI}=\frac{\mathrm{C }12:0+\left(4\times \mathrm{C }14:0\right)+\mathrm{C }16:0}{\mathrm{MUFA}+\mathrm{PUFA}}$$

2 $$\mathrm{TI}=\frac{\mathrm{C }14:0+\mathrm{C }16:0+\mathrm{C }18:0}{0.5\times \mathrm{MUFA}+0.5\times \mathrm{PUFA n}-6+\left(3\times \mathrm{PUFA n}-3\right)+\left(\frac{\mathrm{PUFA n}-3}{\mathrm{PUFA n}-6}\right)}.$$

### Positional distribution of fatty acids in triacylglycerols

The samples were prepared and analyzed following the methodology described by Ziarno et al.^34^. In summary, the hydrolysis of TAGs was conducted on the lipid fraction of the tested samples using porcine pancreas lipase (Merck KGaA), which has activity against ester bonds in the *sn*–1 and *sn*–3 positions of TAGs. To separate the hydrolysis products of triacylglycerols, chromatographic plates (Silica on TLC Alu foils from Fluka Analytical) were employed. Subsequently, the separated samples were prepared for analysis using gas chromatography to determine the positioning of individual fatty acids at specific triacylglycerol (TAG) positions. The proportions of fatty acids in the *sn*-1 and *sn*-3 positions were calculated using Eq. (3):

3 $$\mathrm{sn}-\mathrm{1,3}=\frac{3\times\mathrm{(FA\, in\, TAGs)}-(\mathrm{FA\, in\, sn-2\, MAG})}{2},$$

where: *sn*-1,3 – are the shares of fatty acid in the *sn*-1 and *sn*-3 positions [%]; FA in TAGs—is the share of a fatty acid in the starting triacylglycerols [%]; and FA in *sn*–2 MAG—is the share of a fatty acid in the *sn*–2 position in the final monoacylglycerols [%].

The composition of the *sn*-2 fatty acids compared with the total specific fatty acid share in all positions was determined according to Eq. (4):

4 $$\mathrm{sn}-\mathrm{2}=\frac{(\mathrm{FA\, in\, sn-2\, MAG}) }{3\times\mathrm{(FA\, in\, TAGs})}\times\mathrm{100\%},$$

where: *sn*–2—is the share of a fatty acid in the *sn*-2 position [%]; FA in *sn*-2 MAG—is the share of a fatty acid in the *sn*-2 position in the final monoacylglycerols [%]; and FA in TAGs—is the share of a fatty acid in the starting triacylglycerols [%]. The analysis was performed on six independent samples.

### Statistical analysis

The results are presented as the mean ± standard deviation of the mean (SD) of three independent experiments. Statistical analysis was performed using the Statgraphics Centurion XVI program (The Plains, Virginia, USA). A *p-*value < 0.05 was considered statistically significant. One-way analysis of variance (ANOVA) was employed for the analysis. Additionally, correlation coefficients were calculated and a principal components analysis (PCA) was conducted using a correlation matrix. To interpret the fatty acid profile results and atherogenic and thrombogenic indices. a cluster analysis was performed using positions of the samples at the first and second dimension, employing a dissimilarity matrix and Ward method.

## Results and discussion

### Basic chemical and microbiological composition of the processed milk and the rennet cheese samples produced with the starter cultures tested

The fresh milk used to obtain all cheese samples had an average dry matter content of 12.8 ± 0.10%, comprising 3.62 ± 0.07% protein and 3.76 ± 0.17% fat (Table 1). The cheese samples obtained differed in their chemical composition, including water, protein, and fat content, depending on the microorganisms used and the cheese maturation time. As the cheese ripening time increased, a statistically significant reduction in water content was observed, with the most substantial effect seen in cheese samples of the variant obtained using a typical mesophilic cheese culture and a monoculture of *L. casei* (KMC matured). Consequently, the average protein content increased, but statistically significant changes were observed only for cheese samples of the variant obtained using a typical mesophilic cheese culture and a monoculture of *Propionibacterium* (KMP matured). Interestingly, in the case of fresh cheese samples, only the cheeses of the variant obtained using a typical mesophilic cheese culture and *L. casei* monoculture (KMC fresh) had significantly different protein contents compared with other fresh cheese samples. The fat content also significantly distinguished the fresh cheese samples from the mature ones. When expressing the fat content as a percentage of dry matter of the cheese samples, mature cheese samples of variants were obtained using typical mesophilic cheese culture and *L. casei* monoculture (KMC matured). The matured samples were found to contain significantly less fat in dry matter (d.m.) than fresh cheese samples. Regarding salt content, there were no statistically significant differences among the cheese samples, and the average sodium chloride content of the received samples range from of 1.93% to 1.94%. The acidity (pH) of the cheese samples immediately after receiving them, fell within the range of 5.60–5.86, regardless of the variant of cheese received. Moreover, the maturation process caused a statistically significant increase in acidity (reduction in pH values) to levels already dependent on the variant of cheese received. The greatest reduction in pH values was observed for cheese samples of variants obtained using typical mesophilic cheese culture and *L. casei* monoculture (KMC matured), those obtained using typical mesophilic cheese culture and *Propionibacterium* monoculture (KMP matured), and those obtained using typical mesophilic cheese culture and *L. casei* and *Propionibacterium* monocultures (KMCP matured).

**Table 1:** Basic chemical and microbiological composition of the processing milk and the rennet cheese samples produced with the starter cultures tested (*n* = 6).

Parameter studied	Milk	KM fresh	KMC fresh	KMP fresh	KMCP fresh	KM matured	KMC matured	KMP matured	KMCP matured
pH	6.66^d^ ± 0.01	5.60^c^ ± 0.12	5.61^c^ ± 0.16	5.86^c^ ± 0.10	5.68^c^ ± 0.03	4.88^b^ ± 0.02	4.23^a^ ± 0.02	4.44^a^ ± 0.12	4.33^a^ ± 0.12
water content [%]	87.20^d^ ± 0.10	40.67^c^ ± 0.10	41.73^c^ ± 0.58	42.17^c^ ± 1.42	41.32^c^ ± 0.29	34.36^b^ ± 0.28	31.53^a^ ± 0.12	33.83^a,b^ ± 0.29	32.93^b^ ± 1.04
protein content [%]	3.62^a^ ± 0.07	18.30^b^ ± 0.02	20.42^d,e^ ± 0.06	18.89^b,c^ ± 0.79	19.48^b,c,d^ ± 0.25	20.29^c,d^ ± 0.46	21.78^e^ ± 0.33	20.84^d,e^ ± 0.91	20.86^d,e^ ± 0.62
fat content [%]	3.76^a^ ± 0.17	29.75^b,c^ ± 0.25	30.47^b,c^ ± 0.55	29.45^b^ ± 0.23	30.37^b,c^ ± 0.55	33.00^d^ ± 0.50	32.50^d^ ± 0.00	32.50^d^ ± 0.00	32.50^d^ ± 0.00
fat in d.m. [%]	29.35^a^ ± 1.48	50.14^c,d,e^ ± 0.47	52.29^e^ ± 1.05	50.94^d,e^ ± 0.86	51.75^e^ ± 0.82	50.28^c,d,e^ ± 0.70	47.47^b^ ± 0.09	49.12^b,c,d^ ± 0.21	48.47^b,c^ ± 0.76
salt content [%]	0.69^a^ ± 0.52	1.94^b^ ± 0.01	1.94^b^ ± 0.01	1.93^b^ ± 0.00	1.93^b^ ± 0.01	1.94^b^ ± 0.00	1.93^b^ ± 0.01	1.93^b^ ± 0.01	1.93^b^ ± 0.01
lactococci [log CFU/g]	–	7.9^a,b^ ± 0.7	8.2^a,b^ ± 0.4	8.6^b^ ± 0.1	8.7^b^ ± 0.2	8.5^b^ ± 0.7	7.3^a,b^ ± 0.7	7.2^a,b^ ± 0.5	7.0^a^ ± 0.5
*L. casei* [log CFU/g]	–	–	8.0^a^ ± 0.5	–	8.6^a^ ± 0.1	–	8.5^a^ ± 0.9	–	7.7^a^ ± 0.1
*Propionibacterium* [log CFU/g]	–	–	–	8.4^b^ ± 0.3	8.5^b^ ± 0.1	–	–	7.3^a^ ± 0.2	7.4^a^ ± 0.3

–Not determined.

Explanation of sample codes: KM—samples made with F-DVS Flora Danica; KMC—samples made with F-DVS Flora Danica + F-DVS L. casei-01; KMP—samples made with F-DVS Flora Danica + PROPIONICI; KMCP—samples made with F-DVS Flora Danica + F-DVS L. casei-01 + PROPIONICI; fresh”—samples fresh; “matured”—samples after 6th weeks of ripening.

a,b,c,…—the same letter indices within the same line do not mean statistically significant differences at the significance level of 0.05.

The population of total lactococci in the fresh cheeses initially ranged from 7.9 to 8.7 log CFU/g. During the cheese ripening process, these lactic acid bacteria significantly decreased in two cheese variants: those obtained using a typical mesophilic cheese culture (KM matured) and those obtained using a typical mesophilic cheese culture with monocultures of *L. casei* and *Propionibacterium* (KMCP matured). However, the population of *L. casei* remained stable during cheese ripening, with all cheese samples containing this monoculture used as an additive (KMC and KMCP samples), ranging from 7.7 to 8.6 log CFU/g. On the other hand, the population of *Propionibacterium* in all cheese samples with this monoculture additive (KMP and KMCP samples) varied depending on the cheese maturation time. In fresh cheese samples, the population of the bacteria in question was in the range of 8.4–8.5 log CFU/g and statistically reduced to 7.3–7.4 log CFU/g after maturation.

No *Enterobacteriaceae,* mold or yeast was found in any of the cheese samples obtained.

In the cheeses obtained in this study, the predominant microorganisms were lactococci, whereas the accessory microorganisms were monocultures of *L. casei* and *Propionibacterium*. Hoier^35^ stresses the potential therapeutic value of dairy products containing intestinal microbiota depends on the presence of sufficient active cells at the time of consumption. Ryhanen et al.^36^ state that for a cheese to be considered probiotic, the population of probiotic lactic acid bacteria should ideally be at least 6 log CFU/g. As discussed earlier, during the entire ripening period of the cheeses obtained in the present study, the populations of total lactococci, *L. casei* and *Propionibacterium* were well above the level of 6 log CFU/g but decreased with time. The survival of lactococci cells observed during cheese ripening in this study is consistent with the findings of Bzducha and Obiedziński^37^. Additionally, Gomes et al.^38^ reported a reduction in lactobacilli during 9 weeks of ripening of Cheddar cheese by as much as two logarithmic orders. Lactobacilli such as *L. rhamnosus, L. casei* and L. *paracasei* showed high survival during Cheddar cheese ripening for 32 weeks, reaching an abundance of 7–8 log CFU/g. Low viability of lactobacilli in rennet cheese was also reported by Godward and Kailasapathy^39^ with the population of lactobacilli never reaching the recommended therapeutic minimum. The vital functions of microorganisms are influenced by the presence of water and the concentration of compounds soluble in it (salts and saccharides). Optimal water activity is essential for bacterial growth, whereas decreased water activity during cheese ripening hinders the growth and metabolic activity of starter cultures. In the first stage of cheese production, water activity is 0.99 and contributes to bacterial growth and starter culture activity. The addition of salt and the hydrolysis of proteins, amino acids, and triglycerides during ripening contribute to the decrease in water activity. During ripening, water activity decreases to a level of 0.917–0.988^40^, impacting the optimal conditions for the growth of starter cultures and affecting their metabolic activity and growth. Thus, water activity contributes to the control of their metabolic activity and growth. However, Stanton et al.^41^ noted that during the ripening of rennet cheeses, increased acidity and high-fat content protect microorganisms during their passage through the human gastrointestinal tract, allowing for the use of new cultures of cheese-making bacteria with a high survival rate in its environment, i.e., lactobacilli such as *L. paracasei* and *L. salivarius*. Moreover, Bzducha and Obiedziński^37^ reported a decrease in the survival rate of *L. casei* bacterial cells at week 2 of ripening indicating that high bacterial survival in cheese is influenced by on the bacterial strain used, metabolic interactions, fermentation and storage conditions, and pH^42–44^.

### Free lysine concentration

**Figure 1:**
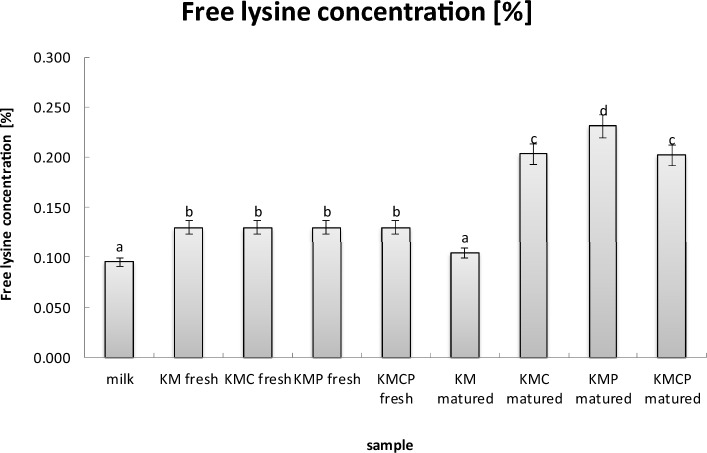
Free lysine concentration [%]. Values are presented as the mean ± SD (*n* = 6).

The results obtained for the fresh cheese samples did not show significant differences among each other. However, the fresh milk and mature cheese samples of the variant obtained using a typical mesophilic cheese culture (KM matured) had the lowest content of available lysine compared with other tested samples. On the other hand, the statistically highest content of available lysine was found in samples of mature cheese variants obtained using a typical mesophilic cheese culture and *Propionibacterium* monoculture (KMP matured) (Fig. 1). Following closely were the samples of mature cheese variants obtained using a typical mesophilic cheese culture and *L. casei* monoculture (KMC matured) or a combination of *L. casei* and *Propionibacterium* monocultures (KMCP matured).

Therefore, it can be concluded that the content of available lysine in the cheeses is influenced by the type of bacterial primers used, and the maturation time of the milk matrices, which affects the metabolic activity of the lactic acid bacteria used in the process. The results indicate that a properly used bacterial mixture influences a higher content of amino acid compared with a single culture, as confirmed by studies conducted by Ciriello et al.^45^ and Moreira et al.^46^. From the cited literature the fact is known that the proteolysis and the amount of free lysine increase with the aging time of the cheese, while the different activity associated with different strains of lactic acid bacteria or propionic bacteria represents an interesting new aspect of the present study.

### Free glycine content

**Figure 2:**
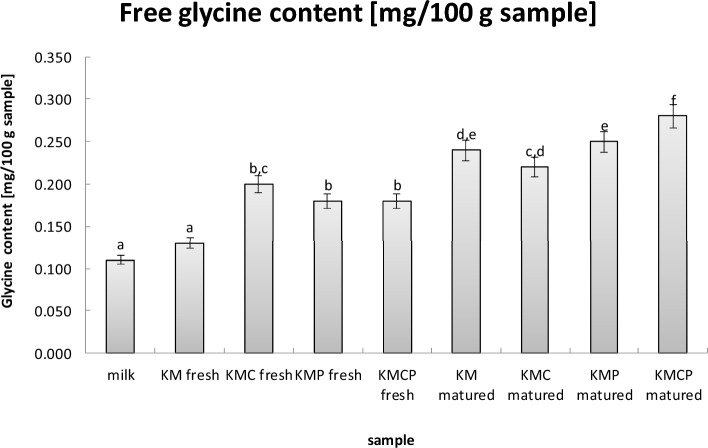
Free glycine content [mg/100 g sample]. Values are presented as the mean ± SD (*n* = 6).

There were statistically significant differences in free glycine content between the samples of fresh milk and fresh cheese (Fig. 2). The addition of *Propionibacterium* monoculture and/or *L. casei* monoculture resulted in a significantly higher free glycine content compared to the fresh milk and fresh cheese samples obtained using a typical mesophilic cheese culture (KM fresh).  For the cheeses examined after a 6-week maturation period, the highest concentration of free glycine was recorded in cheese samples obtained using all three starter cultures (KMCP matured). A slightly lower concentration of free glycine was found in cheese samples obtained using typical mesophilic cheese culture (KM matured) and a *Propionibacterium* monoculture (KMP matured). Thus, it was proven that the addition of *Propionibacterium* monocultures has a significantly stronger influence on the free glycine content in the matured cheese samples than the addition of *L. casei* monocultures. The study by Murgia et al.^47^, analyzing the quality of Sardinian cheese, further confirmed that the amino acid content changed during ripening, confirming the results of the present study. The observed phenomenon can be explained by the increasing the activity of lactic acid bacteria after they adapt to the conditions and initiate fermentation, leading to the actual process of proteolysis. This explanation is in line with the findings of the present study. The composition of the starter bacteria can affect the level of amino acids released to varying degrees, based on the enzymatic system, and the autolysis process itself in the cheese^46,48,49^. Moreira et al.^46^ confirmed possible differences in protein digestibility depending on the type of microorganisms used in the Gorgonzola-type cheese. Williams et al.^50^ also examined the metabolic activity of lactic acid bacteria in Cheddar cheese and confirmed interspecific differences between lactobacilli and lactococci in the ability to catalyze protein hydrolysates and single amino acids.

### Free methionine concentration

**Figure 3:**
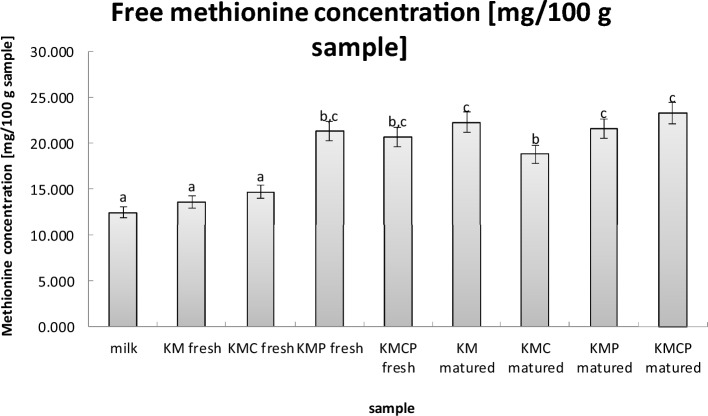
Free methionine concentration [mg/100 g sample]. Values are presented as the mean ± SD (*n* = 6).

The lowest methionine values were detected in fresh cheese samples of variants obtained using a typical mesophilic cheese culture (KM fresh) or a combination of typical mesophilic cheese culture and *L. casei* monoculture (KMC fresh), similar to the methionine content comparable with that of amino acid content present in fresh milk samples (Fig. 3). The highest free methionine content was found in samples of mature cheese variants obtained using typical mesophilic cheese culture (KM matured) and *Propionibacterium* monoculture (KMP matured and KMCP matured), surpassing samples of mature cheese variants obtained using typical mesophilic cheese culture and *L. casei* monoculture (KMC matured).

Therefore, it can be concluded that the content of free methionine in rennet cheeses can not only be affected by factors such as maturation time, but also by the type of microorganisms used. This is supported by previous studies conducted by other researchers^51,52^. Each of the lactic streptococci, lactobacilli and propionic bacteria produces an enzyme specific to itself that can have varying effects on hydrolysis, thus affecting the final content of the free amino acid in rennet cheeses^51^. The conclusions of the work also include the interstrain interactions that occur between the lactococci, lactobacilli and propionibacteria present in cheese, which can also be related to the results of Miks-Krajnik^51^. Baer^53^ demonstrated, that the addition of rennet to cheese milk stimulated the activity of propionibacteria, whereas their growth was dependent on the primary hydrolysis of casein. Meanwhile, the lactic acid bacteria starter culture influenced the growth of propionibacteria in milk. Interestingly, Baer^53^ observed that the rennet added to cheese milk did not provoke the growth of propionibacteria and lactic acid bacteria but produced a significant increase in free amino acids. This indicates the impact of rennet and lactic acid bacteria starter culture on the kinetic of enzymatic (proteinase) activity of propionibacteria.

### Fatty acid profile of fat of the rennet cheese samples produced with the starter cultures tested

Table 2 presents the results of fatty acid content in the fresh milk sample and all variants tested before and after six weeks of the ripening process. In both fresh milk and cheese samples of all variants, saturated fatty acids (SFAs) dominated (11 different SFAs were identified, but the proportion of most of them was below the 2% level). Palmitic acid (C 16:0) was the most abundant in each case analyzed (its percentage share was at the level of 30%), whereas myristic (C 14:0) and stearic (C 18:0) acids ranked next (their share in the lipid phase was in the range of 10–12%). These findings align with the literature data^54,55^. Comparing the SFA profile of fresh milk samples and cheese samples before and after the ripening process, statistically significant differences in the share of lauric (12:0), myristic (C 14:0), and stearic (C 18:0) acids were observed. The highest share of SFAs in the fatty acid pool was identified in cheese samples of the KMP fresh variant, while the lowest share of lauric acid (12:0) in the pool of fatty acids was recorded in the samples of cheeses of the KMCP matured variant, the lowest share of myristic acid (C 14:0) in the fatty acid pool was recorded in cheese samples of KM matured, KMC matured or KMCP matured variants, and the lowest share of stearic acid (C 18:0) in the fatty acid pool was recorded in cheese samples of the KM matured, KMC matured and KMCP matured variants*.* These results indicate that the addition of *L. casei* monoculture has a more significant effect on the SFA share in the fatty acid pool compared with *Propionibacterium* monoculture in the lactic acid bacteria starter culture.

**Table 2:** Fatty acid profile [% of total fatty acids] of fat of the rennet cheese samples produced with the starter cultures tested (*n* = 6).

Fatty acid	Milk	KM fresh	KMC fresh	KMP fresh	KMCP fresh	KM matured	KMC matured	KMP matured	KMCP matured
C4:0	2.33^a^ ± 0.02	2.47^a^ ± 0.50	1.90^a^ ± 0.08	2.37^a^ ± 0.27	2.28^a^ ± 0.06	2.07^a^ ± 0.06	2.24^a^ ± 0.08	2.10^a^ ± 0.06	2.36^a^ ± 0.09
C6:0	1.88^a^ ± 0.11	2.00^a^ ± 0.49	1.63^a^ ± 0.08	1.97^a^ ± 0.20	1.85^a^ ± 0.07	1.59^a^ ± 0.23	1.77^a^ ± 0.09	1.79^a^ ± 0.08	1.87^a^ ± 0.09
C8:0	1.39 ^a^ ± 0.15	1.46^a^ ± 0.34	1.19^a^ ± 0.10	1.42^a^ ± 0.17	1.29^a^ ± 0.08	1.14^a^ ± 0.13	1.21^a^ ± 0.12	1.27^a^ ± 0.10	1.27^a^ ± 0.08
C10:0	3.01 ^a^ ± 0.07	3.27^a^ ± 0.75	2.85^a^ ± 0.04	3.34^a^ ± 0.22	3.00^a^ ± 0.04	2.51^a^ ± 0.43	2.79^a^ ± 0.17	3.04^a^ ± 0.05	2.72^a^ ± 0.05
C12:0	3.54 ^a,b^ ± 0.13	3.92^a,b^ ± 0.84	3.59^a,b^ ± 0.03	4.16^b^ ± 0.20	3.69^a,b^ ± 0.03	3.27^a,b^ ± 0.40	3.34^a,b^ ± 0.22	3.63^a,b^ ± 0.06	2.99^a^ ± 0.19
C14:0	10.80 ^a,b^ ± 0.25	11.36^a,b^ ± 1.77	11.13^a,b^ ± 0.26	12.46^b^ ± 0.22	11.24^a,b^ ± 0.21	10.55^a^ ± 0.51	10.58^a^ ± 0.33	11.12^a,b^ ± 0.22	10.06^a^ ± 0.12
C14:1	1.44 ^b,c^ ± 0.23	1.52^c^ ± 0.22	1.43^a^ ± 0.08	1.59^c^ ± 0.10	1.44^b,c^ ± 0.08	1.06^a,b^ ± 0.06	1.02^a^ ± 0.13	1.06^a,b^ ± 0.14	0.97^a^ ± 0.11
n.i.1	0.66^a^ ± 0.17	0.70^a^ ± 0.06	0.61^a^ ± 0.11	0.67^a^ ± 0.11	0.61^a^ ± 0.11	0.52^a^ ± 0.15	0.57^a^ ± 0.12	0.58^a^ ± 0.11	0.59^a^ ± 0.10
C15:0	1.39^a^ ± 0.19	1.39^a^ ± 0.13	1.33^a^ ± 0.09	1.43^a^ ± 0.08	1.33^a^ ± 0.09	1.25^a^ ± 0.06	1.23^a^ ± 0.11	1.26^a^ ± 0.11	1.28^a^ ± 0.08
C15:1	0.36^a^ ± 0.29	0.37^a^ ± 0.02	0.30^a^ ± 0.12	0.31^a^ ± 0.12	0.30^a^ ± 0.12	0.34^a^ ± 0.01	0.26^a^ ± 0.13	0.27^a^ ± 0.12	0.28^a^ ± 0.12
C16:0	29.98^a^ ± 1.21	30.28^a^ ± 0.28	30.69^a^ ± 0.90	31.36^a^ ± 0.95	30.96^a^ ± 0.89	31.24^a^ ± 0.80	31.04^a^ ± 0.84	30.84^a^ ± 0.66	30.99^a^ ± 0.96
C16:1	2.30^a^ ± 0.24	2.33^a^ ± 0.05	2.33^a^ ± 0.06	2.36^a^ ± 0.05	2.32^a^ ± 0.06	2.19^a^ ± 0.12	2.14^a^ ± 0.12	2.23^a^ ± 0.08	2.32^a^ ± 0.06
n.i.2	0.77^a^ ± 0.21	0.80^a^ ± 0.03	0.79^a^ ± 0.10	0.77^a^ ± 0.10	0.79^a^ ± 0.10	0.66^a^ ± 0.17	0.72^a^ ± 0.13	0.73^a^ ± 0.09	0.81^a^ ± 0.09
C17:0	0.75^a^ ± 0.22	0.74^a^ ± 0.08	0.74^a^ ± 0.10	0.66^a^ ± 0.12	0.69^a^ ± 0.10	0.65^a^ ± 0.16	0.76^a^ ± 0.10	0.74^a^ ± 0.10	0.75^a^ ± 0.11
C17:1	0.48^a^ ± 0.23	0.45^a^ ± 0.04	0.38^a^ ± 0.12	0.36^a^ ± 0.11	0.39^a^ ± 0.11	0.29^a^ ± 0.22	0.35^a^ ± 0.13	0.36^a^ ± 0.12	0.40^a^ ± 0.12
C18:0	10.95^a,b^ ± 0.20	10.19^a,b^ ± 1.52	11.17^a,b^ ± 0.16	9.77^a^ ± 0.32	11.20^a,b^ ± 0.36	11.94^b^ ± 0.63	11.70^b^ ± 0.42	11.30^a,b^ ± 0.30	11.92^b^ ± 0.30
C18:1 n-9t	1.66^a,b^ ± 0.23	1.57^a,b^ ± 0.07	1.77^b^ ± 0.08	1.85^b^ ± 0.08	0.69^a^ ± 1.01	3.92^c^ ± 0.13	3.04^c^ ± 0.12	3.55^c^ ± 0.18	1.73^b^ ± 0.09
C18:1 n-9c	22.93^a^ ± 1.15	21.93^a^ ± 3.23	22.88^a^ ± 0.48	20.40^a^ ± 0.82	22.65^a^ ± 0.51	21.65^a^ ± 1.09	22.02^a^ ± 0.79	21.04^a^ ± 0.85	23.18^a^ ± 0.69
C18:2 n-6t	0.41^a^ ± 0.19	0.34^a^ ± 0.06	0.30^a^ ± 0.12	0.24^a^ ± 0.12	0.30^a^ ± 0.12	0.15^a^ ± 0.11	0.15^a^ ± 0.12	0.14^a^ ± 0.12	0.26^a^ ± 0.12
C18:2 n-6c	2.02^a,b^ ± 0.06	1.96^a,b^ ± 0.29	2.06^a,b^ ± 0.12	1.73^a^ ± 0.07	2.07^a,b^ ± 0.13	2.08^a,b^ ± 0.05	2.15^b^ ± 0.06	2.08^a,b^ ± 0.06	2.24^b^ ± 0.06
C18:3 n-3	0.47^a^ ± 0.25	0.42^a^ ± 0.06	0.38^a^ ± 0.12	0.32^a^ ± 0.12	0.38^a^ ± 0.11	0.37^a^ ± 0.10	0.36^a^ ± 0.12	0.35^a^ ± 0.12	0.42^a^ ± 0.11
C20:0	0.49^a^ ± 0.15	0.53^a^ ± 0.09	0.55^a^ ± 0.11	0.45^a^ ± 0.11	0.55^a^ ± 0.10	0.57^a^ ± 0.09	0.55^a^ ± 0.11	0.53^a^ ± 0.11	0.58^a^ ± 0.11
SFA (total)	66.50 ± 0.24	67.60 ± 0.62	66.77 ± 0.18	69.40 ± 0.26	68.08 ± 0.18	66.78 ± 0.32	67.22 ± 0.23	67.62 ± 0.17	66.78 ± 0.20
MUFA (total)	29.16 ± 0.39	28.18 ± 0.60	29.08 ± 0.16	26.86 ± 0.21	27.78 ± 0.32	29.44 ± 0.27	28.83 ± 0.29	28.50 ± 0.25	28.89 ± 0.20
PUFA (total)	2.91 ± 0.17	2.72 ± 0.14	2.74 ± 0.12	2.29 ± 0.10	2.74 ± 0.12	2.60 ± 0.09	2.66 ± 0.10	2.57 ± 0.10	2.92 ± 0.11
AI	2.39 ± 0.10	2.62 ± 0.58	2.40 ± 0.12	2.48 ± 0.10	2.44 ± 0.07	2.93 ± 0.08	2.54 ± 0.07	2.61 ± 0.13	2.33 ± 0.08
TI	2.98 ± 0.25	3.13 ± 0.39	3.14 ± 0.10	3.12 ± 0.18	3.17 ± 0.14	3.45 ± 0.17	3.22 ± 0.14	3.27 ± 0.23	3.10 ± 0.19

n.i. – unidentified.

Explanation of sample codes: KM—samples made with F-DVS Flora Danica; KMC—samples made with F-DVS Flora Danica + F-DVS L. casei-01; KMP—samples made with F-DVS Flora Danica + PROPIONICI; KMCP—samples made with F-DVS Flora Danica + F-DVS L. casei-01 + PROPIONICI; fresh”—samples fresh; “matured”—samples after 6th weeks of ripening.

a,b,c,… – the same letter indices within the same line do not mean statistically significant differences at the significance level of 0.05.

The second group of fatty acids present in the fat of fresh milk and cheese samples, before or after the ripening process, consisted of monounsaturated fatty acids (MUFAs), with oleic acid (C 18:1 *n*-9c) being the predominant MUFA (exceeding the 20% in all cheese samples). The share of (9Z)-tetradecanoic (C 14:1) and elaidic (C 18:1 *n*-9t) acids in the fatty acid pool statistically differentiated the milk and cheese samples. The lowest share of (9Z)-tetradecanoic acid (C 14:1) in the fatty acid pool was recorded in cheese samples of KMC matured and KMCP matured variants, whereas the lowest share of MUFAs in the fatty acid pool was identified in cheese samples of the KMP fresh variant. These findings indicate a variable lipolytic activity of the added *L. casei* and *Propionibacterium* monocultures to the lactic acid bacteria starter culture, influencing the monounsaturated fatty acid profile in the cheese.

Polyunsaturated fatty acids (PUFAs) were also identified in the fat of fresh milk samples and cheese samples, accounting for 2–3% of the fatty acid pool. The share of conjugated linoleic acid (CLA) in the fatty acid pool significantly differentiated the fresh milk samples and cheese samples. The highest share of CLA in the fatty acid pool was recorded in the cheese samples of the KMC fresh or KMCP matured variants, whereas the lowest share of CLA was identified in the cheese samples of the KMP fresh samples. Similarly, the lowest share of total PUFAs in the fatty acid pool was identified in cheese samples of the KMP fresh variant.

Rodrigues et al.^56^ demonstrated that the incorporation of specific bacterial strains can influence the functional parameters of cheeses, including their fatty acid profiles. For instance, the addition of *L. casei* resulted in a decrease in stearic acid content during the 60-day ripening of cheeses. Furthermore, the cited researchers observed an increase in the content of certain acids, including CLA, ALA and GLA isomers during the ripening of cheeses, probably caused by the greater lipolytic activity of the bacteria added to the products.

Numerous studies have indicated that lactic acid bacteria strains can produce CLA through the isomerization of linoleic acid (LA) using the linoleate isomerase enzyme^57–60^. During the Van Nieuwenhove et al.^61^ experiment, changes in CLA content were observed during cheese ripening. Cheeses made with *Lactococcus lactis* subsp. *lactis* R-603 and *Bifidobacterium lactis* Bb-12 were used as model products. Cheeses ripened at 6 °C for 8 weeks and showed no significant changes in CLA. However, cheeses with *Bifidobacterium lactis* Bb-12 that were ripened at a higher temperature (14 °C) were found to have an increase in CLA during the eighth week of ripening. This increase could be attributed to the lipolytic activity of lactic acid bacteria, potentially mediated by lipases and esterases, which are intracellular enzymes released during cell lysis over time^62^. Zaręba^62^ demonstrated that storage time significantly influenced the final content of dominant fatty acids in the lipid phase of soy yogurts fermented with a variety of lactic acid bacteria.

### Atherogenic and thrombogenic indices

Furthermore, based on fatty acid composition, the AI and TI were determined to provide information on the risk of cardiovascular disease^33^. The highest AI value observed for cheese samples of the KM matured variant (with a mean value of 2.93 ± 0.08), primarily due to the different proportions of long-chain SFAs and unsaturated fatty acids compared with other cheese and fresh milk samples. On the other hand, the lowest AI values were achieved for fresh milk samples (with a mean value of 2.39 ± 0.10) and matured cheese samples of the KMCP matured variant (with a mean value of 2.33 ± 0.08). These AI values are comparable with those reported by Peña-Serna et al.^30^ for cow's milk lipids (with a mean value of 2.8).

**Figure 4:**
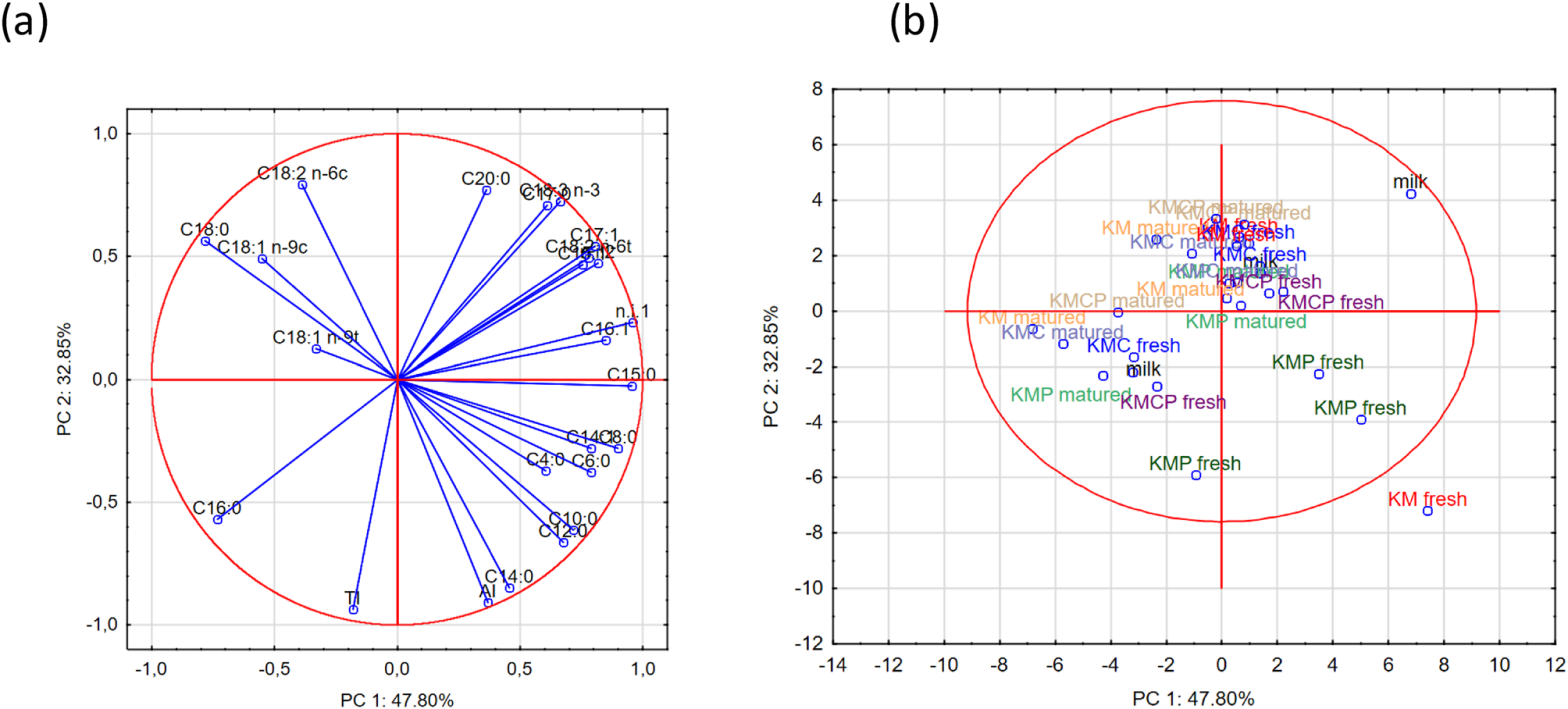
Dispersion of attributes on a surface (**a**) and projection of samples (**b**) on a surface for the first two principal components (PC).

**Figure 5:**
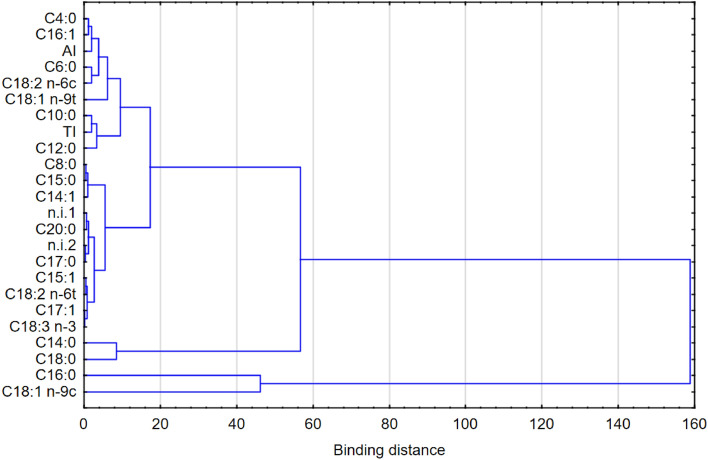
Tree diagram. Cluster analysis of variables of the rennet cheese samples produced with the starter cultures tested.

The PCA conducted to analyze the data revealed 24 factors influencing the fatty acid profile of the rennet cheese samples produced with the tested starter cultures. The first two factors, PC1 and PC2, explained 80.65% of the variable variances. The dispersion of these factors is illustrated in Fig. 4. The diagram clearly demonstrates a strong correlation between the AI and specific SFAs, including C 14:0, C 12:0, C 10:0, C 6:0, C 4:0, C 8:0, and C 14:1. Higher levels of C 14:0 fatty acid in the cheese samples were associated with an increased AI. On the opposite side of the axis, vectors representing fatty acids such as C 18:0, C 18:1 *n*-9c, C 18:1 *n*-9t, and C 18:2 *n*-6c were correlated in an opposite manner with the AI. This suggests that the addition of *L. casei* monoculture to the lactic acid bacteria starter culture had a more significant effect on the AI compared with the addition of *Propionibacterium* monoculture. A strong correlation between TI and C 16:0 was also evident. On the opposite side of the axis, vectors representing fatty acids including C 20:0, C 18:3 *n*-3, C 17:0, C 17:1, C 15:1, C 18:26t, and C 16:1 showed an opposite correlation with the TI. The cluster analysis (Fig. 5) further confirmed the relationships between individual variables and their strengths. Two distinct clusters were identified. The first cluster was related to the AI and TI indices and strongly linked to fatty acids such as C 4:0, C 16:1, C 6:0, C 18:2 *n*-6c, C 18:1 *n*-9t, and C 10:0. This reinforces the significant impact of the added *L. casei* or *Propionibacterium* monocultures on the AI and TI indices. The second cluster encompassed the other remaining fatty acids.

### Percentage of fatty acids found in triacylglycerols, in sn-2 positions and in sn-1,3 positions of triacylglycerols in the fat of the rennet cheese samples produced with the starter cultures tested

The study further explores the impact of triacylglycerols structure and composition on the functional properties of fat, which can ultimately lead to the production of more beneficial products for the body^63^. The present study utilizes the property of pancreatic lipase capable of cleaving acyl residues at the outer positions (*sn*-1 and *sn*-3) (Table 3). This property is used to isolate the inner positions individually, whereas the outer positions are treated as equimolar. Myristic acid (C 14:0), palmitic acid (C 16:0), stearic acid (C 18:0), and acid oleic (C 18:1 *n*-9c) were the four most abundant fatty acids analyzed in TAGs. For this reason, the values shown in Table 3 do not add up to 100% in each column. For each of the fresh milk and cheese samples analyzed, the trend regarding the share of the fatty acids in question was the same. The highest share of palmitic acid (C 16:0) was observed in all samples, but it did not significantly differentiate the studied samples. Oleic acid (C 18:1 *n*-9c) showed the second-highest share in TAG significantly differentiated the samples, whereas the lowest share was recorded in cheese samples of the KM fresh, the KMP fresh, and KMP matured variants. On the other hand, myristic acid (C 14:0) and stearic acid (C 18:0) proportions in TAG were similar in level but varied significantly among the samples. The highest proportion of myristic acid in TAG was found in cheese samples of the KMP fresh variant, whereas the lowest share of myristic acid in TAG was found in cheese samples of the KMCP matured. The highest share of stearic acid in TAG was found in cheese samples of the KMC matured or KMCP matured variants. The lowest share of stearic acid in TAG was found in the cheese samples of the KM fresh or KMP fresh variants. These changes confirm the lipolytic or esterolytic activities of the *L. casei* monoculture and/or *Propionibacterium* monoculture added to the lactic acid bacteria of starter cultures.

**Table 3:** Percentage of fatty acids [% of total fatty acids] found in triacylglycerols, in *sn*-2 positions and in *sn*-1,3 positions of triacylglycerols in the fat of the rennet cheese samples produced with the starter cultures tested (*n* = 6).

Fatty acid	Percentage in TAG [%]								
	Milk	KM fresh	KMC fresh	KMP fresh	KMCP fresh	KM matured	KMC matured	KMP matured	KMCP matured
C14:0	10.9^a,b,c^ ± 0.5	12.0^b,c^ ± 0.6	11.0^a,b,c^ ± 0.6	12.4^c^ ± 0.6	11.2^a,b,c^ ± 0.6	10.7^a,b^ ± 0.5	10.6^a,b^ ± 0.5	11.1^a,b,c^ ± 0.6	10.0^a^ ± 0.5
C16:0	30.1 ^a^ ± 1.5	30.2^a^ ± 1.5	30.2^a^ ± 1.5	30.8^a^ ± 1.5	30.5^a^ ± 1.5	30.8^a^ ± 1.5	30.6^a^ ± 1.5	30.5^a^ ± 1.5	30.5^a^ ± 1.5
C18:0	10.9^a,b^ ± 0.5	9.9^a^ ± 0.5	11.1^a,b^ ± 0.6	9.6^a^ ± 0.5	11.0^a,b^ ± 0.6	11.1^a,b^ ± 0.6	11.5^b^ ± 0.6	11.2^a,b^ ± 0.6	11.8^b^ ± 0.6
C18:1 n-9c	22.9^a,b^ ± 1.1	21.0^a^ ± 1.0	22.7^a,b^ ± 1.1	20.0^a^ ± 1.0	22.4^a,b^ ± 1.1	21.2^a,b^ ± 1.1	21.6^a,b^ ± 1.1	20.6^a^ ± 1.0	24.1^b^ ± 1.2

Fatty acid	Percentage in position sn-2 [%]								
	Milk	KM fresh	KMC fresh	KMP fresh	KMCP fresh	KM matured	KMC matured	KMP matured	KMCP matured
C14:0	15.5^a^ ± 0.8	15.6^a^ ± 0.8	14.3^a^ ± 0.7	15.7^a^ ± 0.8	15.4^a^ ± 0.8	14.6^a^ ± 0.7	15.6^a^ ± 0.8	14.8^a^ ± 0.7	15.9^a^ ± 0.8
C16:0	32.6^a^ ± 1.6	36.0^a^ ± 1.8	34.8^a^ ± 1.7	34.9^a^ ± 1.7	36.8^a^ ± 1.8	35.8^a^ ± 1.8	36.0^a^ ± 1.8	34.7^a^ ± 1.7	35.3^a^ ± 1.8
C18:0	5.6^a^ ± 0.3	6.7 ^b,c,d^ ± 0.3	7.6^d^ ± 0.4	6.8^b,c,d^ ± 0.3	7.2^c,d^ ± 0.4	7.5^d^ ± 0.4	6.6^b,c,d^ ± 0.3	6.3^a,b,c^ ± 0.3	5.9^a,b^ ± 0.3
C18:1 n-9c	14.8^a^ ± 0.7	18.3^b^ ± 0.9	16.6^a,b^ ± 0.8	17.4^b^ ± 0.9	16.9^a,b^ ± 0.8	18.4^b^ ± 0.9	17.2^a,b^ ± 0.9	16.6^a,b^ ± 0.8	16.8^a,b^ ± 0.8

Fatty acid	Percentage in position sn-1,3 [%]								
	Milk	KM fresh	KMC fresh	KMP fresh	KMCP fresh	KM matured	KMC matured	KMP matured	KMCP matured
C14:0	8.5^b^ ± 0.4	10.2^c,d^ ± 0.5	9.4^b,c^ ± 0.5	10.8^d^ ± 0.5	9.1^b,c^ ± 0.5	8.7^b^ ± 0.4	8.1^a,b^ ± 0.4	9.2^b,c^ ± 0.5	7.1^a^ ± 0.4
C16:0	28.8^a^ ± 1.4	27.3^a^ ± 1.4	27.9^a^ ± 1.4	28.8^a^ ± 1.4	27.3^a^ ± 1.4	28.4 ^a^ ± 1.4	27.9^a^ ± 1.4	28.4^a^ ± 1.4	28.1^a^ ± 1.4
C18:0	13.6^c,d^ ± 0.7	11.5^b,c^ ± 0.6	12.8^a,b,c^ ± 0.6	11.1^a^ ± 0.6	13.0^b,c,d^ ± 0.7	12.8^a,b,c^ ± 0.6	14.0^c,d^ ± 0.7	13.6^c,d^ ± 0.7	14.7^d^ ± 0.7
C18:1 n-9c	27.0^d,e^ ± 1.4	22.3^a,b^ ± 1.1	25.8^c,d,e^ ± 1.3	21.3^a^ ± 1.1	25.1^b,c,d,e^ ± 1.3	22.6^a,b,c^ ± 1.1	23.8^a,b,c^ ± 1.2	22.7^a,b,c^ ± 1.1	27.8^e^ ± 1.4

Explanation of sample codes: KM—samples made with F-DVS Flora Danica; KMC—samples made with F-DVS Flora Danica + F-DVS L. casei-01; KMP—samples made with F-DVS Flora Danica + PROPIONICI; KMCP—samples made with F-DVS Flora Danica + F-DVS L. casei-01 + PROPIONICI; fresh”—samples fresh; “matured”—samples after 6th weeks of ripening.

a,b,c,…—the same letter indices within the same line do not mean statistically significant differences at the significance level of 0.05.

Statistically significant differences were observed in the positioning and preference of the mentioned fatty acids in TAG. Table 3 shows the percentage of fatty acids in *sn*-2 and *sn*-1,3 TAG positions. During the analysis, no fatty acid was observed that had an even distribution in both positions. Myristic acid displayed a preference for the middle position among the FAs analyzed, whereas palmitic acid showed a greater preference for occupying the middle position than the *sn*-1,3 position (its share in the *sn*-2 and *sn*-1,3 TAG positions did not depend on the type of sample studied). Oleic and stearic acids, on the other hand, preferentially occupied the outer position in TAG, with their positioning significantly varying among the different samples, which is also reflected in the results obtained in other literature studies^64^. The highest share of oleic acid in the *sn*-2 TAG position was recorded for cheese samples of KM fresh, KMP fresh, and KM matured variants. The lowest share of oleic acid in the *sn*-2 TAG position was shown in milk samples. Meanwhile, the share of oleic acid in the *sn*-1,3 TAG positions was highest for cheese samples of the KMCP matured variant, whereas it was lowest for cheese samples of the KMP fresh variant. The highest share of stearic acid in the sn-2 TAG position was recorded for cheese samples of the KMC fresh and cheese samples of the KM matured variants. In comparison, the lowest share of stearic acid in the sn-2 TAG position was recorded for milk samples. Meanwhile, the share of stearic acid in the sn-1,3 TAG positions was highest for cheese samples of the KMCP matured variant, whereas it was lowest for cheese samples of the KMP fresh variant.

Milk fatty acid biosynthesis exhibits specificity and selectivity for the esterification of individual fatty acids in each of the three available positions^64^. The arrangement of TAG itself is not random; instead, it is specific to each type of fat^63^. For instance, in milk fat, C 4:0 and C 6:0 acids are predominantly found in the *sn*-3 TAG position, whereas C 14:0 and C 12:0 are esterified in the *sn*-2 TAG position, and C 16:0 occupies the *sn*-1 or *sn*-2 TAG position. Long-chain fatty acids, i.e., C18:0 and C18:1, among others, are in the sn-1 TAG position^64,65^. Our research has demonstrated that the preference of the fatty acid’s positions in TAG varies depending on the type of sample tested. Fatty acids that are arranged in the *sn*-3 position of TAG are more susceptible to lipolysis by pancreatic lipase, displaying up to 10 times greater efficacy in cleaving butyric acid from the triacylglycerol molecule in the *sn*-3 position than SFAs with a position in *sn*-1. However, as the fatty acid chain lengthens, the lipase activity gradually decreases^66^. The positioning of fatty acids in in milk fat’s triacylglycerols not only impacts digestion and lipolytic enzyme activity but also plays a crucial role in influencing the sensory characteristics of dairy products, such as ripened cheeses^67^.

Lipolysis and esterification play essential roles during the ripening process of cheeses, influencing the partial decomposition or modification of triacylglycerols, leading to changes in the distribution of FAs in TAG molecules. The specificity of lipases from the microorganisms used is usually observed in the outer positions of TAG, influencing the final preference for occupied positions. The aspect of shaping the positions occupied in TAG found in mold cheeses and rennet long-maturing cheeses was discussed by Molkentin^68^ Molkentin^68^ also observed differences in this type of cheese when it came to positioning in TAG positioning in this cheese variety, indicating the importance of the microbiological composition of the starter culture. The effect of lactobacilli on the fatty acid profile was studied by Ziarno et al.^34^ in fermented beverages derived from legumes. These studies indicated relationships between the type of microorganisms used and the distribution of fatty acids in TAGs. The beverage samples were fermented with *L. delbrueckii* subsp. *bulgaricus* had a higher share of unsaturated FAs in the outer positions compared with those fermented by *L. helveticus*. The specific activity of certain types of lactic bacteria may be related to transesterification, and the specific action of lipases may be directed at specific FAs or specific groups of them. The differences observed in the present study may also be due to the metabolic capabilities of the bacteria used, whereas their enzymes responsible for hydrolysis may have distinct positions of activity. Despite these discrepancies being small, they highlight the potential differences in the impact on the positioning of FAs in TAG of milk fat^34^. Stereo- and regiospecificity are characteristic features of bacterial enzymes. The characteristics and properties of bacterial enzymes responsible for esterification reactions were presented by Lewandowska et al.^69^. Esterases produced by individual microorganisms are strain-dependent and show specific substrate specificity in terms for acyl residues or the position of FAs in TAG^70,71^.

## Conclusion

The fatty acid composition and the distribution of fatty acids in triacylglycerols in matured cheeses are significantly influenced by maturation time and the choice of starter culture. These changes in the fatty acid profile have health implications, particularly concerning the atherogenic and thrombogenic indices. The lowest AT was observed in mature cheese samples from a variant obtained using a mesophilic cheese culture with the addition of *L. casei* and *Propionibacterium* monocultures. These findings indicate that the fatty acid composition of dairy products can impact the risk of cardiovascular disease incidence.

The study also demonstrated that lactic acid bacteria and propionibacteria have an effect on the content of available free lysine, glycine, and methionine in cheeses. The mature cheeses obtained using a combination of mesophilic lactic acid bacteria, *Propionibacterium,* and *L. casei* exhibited the highest free amino acid content. It was evident that the addition of propionibacteria had a more significant influence on the free amino acid content in matured cheese samples compared with the mesophilic lactic acid bacteria starter culture or the addition of *L. casei* monoculture. The study highlights that mesophilic lactic acid bacteria in the starter culture stimulate the growth of propionibacteria in milk leading to the production of a substantial amount of free amino acids.

However, the authors caution that the interpretation of atherogenic and thrombogenic indicators should be approached with care, although the in vitro studies provide valuable insights, they may not offer conclusive evidence for real-world cheese consumption trials. Human studies, particularly in vivo studies involving human volunteers, are needed to better understand the actual health effects of consuming commercially produced cheeses.

## Data Availability

All data generated or analyzed during this study are included in this published article [and its supplementary information files].
